# Modulation of *Anopheles stephensi* Gene Expression by Nitroquine, an Antimalarial Drug against *Plasmodium yoelii* Infection in the Mosquito

**DOI:** 10.1371/journal.pone.0089473

**Published:** 2014-02-24

**Authors:** Jian Zhang, Shuguang Zhang, Yanyan Wang, Wenyue Xu, Jingru Zhang, Haobo Jiang, Fusheng Huang

**Affiliations:** 1 Department of Pathobiology, The Third Military Medical University, Chongqing, P. R. China; 2 Department of Entomology and Plant Pathology, Oklahoma State University, Stillwater, Oklahoma, United States of America; RWTH Aachen University, Germany

## Abstract

**Background:**

Antimalarial drugs may impact mosquito’s defense against *Plasmodium* parasites. Our previous study showed nitroquine significantly reduced infection of *Anopheles stephensi* by *Plasmodium yoelii*, but the underlying mechanism remains unclear. In order to understand how transmission capacity of *An. stephensi* was affected by nitroquine, we explored the transcriptome of adult females after different treatments, examined changes in gene expression profiles, and identified transcripts affected by the drug and parasite.

**Methodology/Principal Findings:**

We extended massively parallel sequencing and data analysis (including gene discovery, expression profiling, and function prediction) to *An. stephensi* before and after Plasmodium infection with or without nitroquine treatment. Using numbers of reads assembled into specific contigs to calculate relative abundances (RAs), we categorized the assembled contigs into four groups according to the differences in RA values infection induced, infection suppressed, drug induced, and drug suppressed. We found both nitroquine in the blood meal and *Plasmodium* infection altered transcription of mosquito genes implicated in diverse processes, including pathogen recognition, signal transduction, prophenoloxidase activation, cytoskeleton assembling, cell adhesion, and oxidative stress. The differential gene expression may have promoted certain defense responses of *An. stephensi* against the parasite and decreased its infectivity.

**Conclusions/Significance:**

Our study indicated that nitroquine may regulate several immune mechanisms at the level of gene transcription in the mosquito against *Plasmodium* infection. This highlights the need for better understanding of antimalarial drug’s impact on parasite survival and transmission. In addition, our data largely enriched the existing sequence information of *An. stephensi*, an epidemiologically important vector species.

## Introduction

Malaria parasites go through major transitions of differentiation as they cross tissue barriers of the mosquito with dramatic number changes [1]–[2]: between gametocytes and ookinetes, between ookinetes and mature oocysts, and between midgut and salivary gland sporozoites [3]. The most critical bottleneck of *Plasmodium* development occurs during ookinete invasion of the midgut epithelium, prior to oocyst development on the basal lamina [4]. Digestive enzymes and mosquito defense proteins are partly responsible for the parasite loss at this stage [5]–[6]. The latter includes pathogen recognition proteins, serine proteases, phenoloxidases, antimicrobial peptides, and others.

External factors may also affect mosquito susceptibility to parasite infection, including ingested antibodies, subsequent blood feeding, and antimalarial drugs in blood meals. Among them, effects of antimalarial drugs on infectivity of parasites in mosquitoes are well documented. Chloroquine mainly targets *Plasmodium* at the erythrocytic stage. Nitroquine (CI-679 or 2,4-diamino-6-[(3,4-dichlorobenzyl)nitros-amino]quinazoline) caused nuclear and cytoplasmic damage in asexual forms of *P. berghei* and *P. cynomolgi* [7]. While there was no further report on this compound from Western countries, safety and efficacy of nitroquine were tested in rodents, chicken, primates, and humans [8]. Nitroquine is highly effective against *P. yoelii*, *P. gallinaceum*, and *P. cynomolgi* at the erythrocytic and exoerythrocytic stages. It interferes with structure and function of the cytoplasm and nucleus of *P. yoelii* exoerythrocytic forms [9]. The action mechanism of nitroquine involves inhibition of *Plasmodium* DNA and protein synthesis [10]–[11]. After further pharmacological and toxicological tests, nitroquine was used in clinical trials on malaria patients after 1973. In nine provinces and fifteen regions, this drug successfully cured 11,407 patients in the field tests [8]. Nitroquine and chloroquine were equally effective against *P. falciparum*. Chloroquine ingestion by mosquito at the time of blood feeding associates with an increase in parasite numbers in the insect host [12]. In contrast, ingested nitroquine leads to a decrease in *Plasmodium* number and infectivity [13]. Using the model system of *Plasmodium yoelii* and *Anopheles stephensi*, we demonstrated that nitroquine induced transcription of a few mosquito genes encoding pattern recognition receptors, signal transducers, cell adhesion molecules, and oxidative stress proteins.

*An. stephensi* is a major malaria vector in the Indian subcontinent [14]. Rapid development and urbanization in this region has led to increase in the mosquito population resulting in frequent malaria epidemics [15]. While recent malaria outbreaks occurred at a higher frequency, mortality became considerably lower. For example, during 2003, only 1006 of the reported 1.78 million cases in India caused deaths but reasons for the mortality reduction are unclear [16]. As an important disease vector, *An. stephensi* has not yet been intensively investigated at the molecular level, which hinders the elucidation of mechanisms for various physiological processes such as how immunity is possibly affected by nitroquine.

Although generation and analysis of cDNA clones from midgut tissue of adult female *An. stephensi* yielded useful sequences [17], the acquired information is limited by the method used and additional experiments are needed to quantify their mRNA levels in different stages or tissues. Recently, a high throughput method is established to efficiently discover genes along with their expression profiles using next-generation sequencing technology [18]. Without resorting to a reference genome and thereby directly uncover process-related gene expression, the study revealed over 103 differentially regulated defense genes in *Manduca sexta*.

To better understand the impact of antimalarial drugs on mosquito’s defense against *Plasmodium* parasites, we designed a study to discover genes with significantly altered mRNA levels in naïve versus *P. yoelii-*infected adult females of *An. stephensi* fed on nitroquine-treated or -untreated mice by the RNA-Seq approach. We largely increased the throughput of sequencing by adopting the Illumina technology and studied effects of the infection and drug on mosquito gene expression.

## Methods and Materials

### Ethics Statement

This study was carried out in strict accordance with the recommendations in the Guide for the Ethics Committee of Third Military Medical University in China. The protocols involving mice were approved by the committee and performed under anesthesia to minimize suffering. Sodium pentobarbital was used to induce anesthesia.

### Mosquitoes Rearing, Nitroquine Treatment, and P. yoelii Infection

*An. stephensi* (Hor strain) mosquitoes were raised at 24°C, 75% humidity under a 12∶12 light-dark cycle and maintained on a 5% sucrose solution during adult stage. Female BALB/c mice were inoculated intraperitoneally with 200 µl of infected blood containing about 1×10^7^ *P. yoelii (*By265-GFP) parasitized erythrocytes obtained from a donor mouse with about 10% parasitemia. The course of infection was followed by examining Giemsa-stained blood smears prepared from tail blood samples collected at different times after inoculation. When the gametocitaemia reached 1%, a curative dose of nitroquine (12 mg/kg) was administered intragastrically to the mice. At 4 h after the drug treatment, gametocytaemia and parasite exagellation were confirmed. The female mosquitoes were fed on anesthetized mice for 2 h and collected at 24 h after blood feeding. Four-day-old adult females (25 per group) were allowed to blood fed on one of the following four groups: uninfected BALB/c mice treated with nitroquine (12 mg/kg) (UD, uninfected mice treated with nitroquine) for 4 h, *P. yoelii-*infected mice treated with the drug (12 mg/kg) (ID, infected mice treated with nitroquine) for 4 h, uninfected mice treated with buffer without nitroquine (UB, uninfected mice treated with buffer) for 4 h, or *P. yoelii*-infected mice treated with buffer only (IB, infected mice treated with buffer) for 4 h. Unfed mosquitoes were removed from the groups. The oocyst number in midgut was measured at 10 days post-blood feeding, and each assay was done with at least 25 mosquitoes, and the data represent three independent experiments. Difference of infection intensity between groups was analyzed by paired sample t-test (Prism 6.01, GraphPad Software, Inc.).

### RNA Extraction, Library Construction and Sequencing, and Read Assembling

Each of the four groups of female mosquitoes was collected 24 h after blood feeding, washed in ice-cold 95% ethanol to remove cuticle lipids, and rinsed in ice-cold water. All carcasses from the same group were combined, frozen in liquid nitrogen, and ground into fine powder for RNA extraction using TRIzol reagent (Invitrogen). mRNA was separately purified from the total RNA samples (1.0 mg each) by binding to cellulose in Mag-Bind mRNA Enrichment Kit (Omega Bio-Tek). For cDNA synthesis, mRNA was reverse transcribed with MMLV-RT (Promega) in the presence of oligo(dT)_15_ primer. Paired-end libraries were constructed from the four groups (UD, ID, UB, and IB) and sequenced at Macrogen Inc (Korea), following Illumina specifications. The four cDNA pools were sequenced on one lane for 101 cycles from both ends on Illumina GA-IIx HiSeq2000.

### De Novo Assembly of Transcriptomes

Filters were applied to remove low quality reads with >33% N’s (indetermination), >33% A’s from the 5′ end (or T’s from the 3′ end) suggestive of poly-A tail, or >34% nucleotides with low Phred quality scores (<20 *i.e*. 1% error), according to Crawford et al [19]. After adaptor trimming, Velvet [20] was employed to assemble the remaining reads in each of the four libraries at different hash lengths (*k*: 29, 35, 41). All contigs from the three exploratory assemblies were summarized by clustering using CD-HIT [21] to generate four datasets of UD, ID, UB, and IB. The default threshold of 90% was used as identity cutoff. In addition, all retained reads after filtration in the four libraries were assembled at four hash lengths (*k*: 21, 35, 49, 59). All contigs in the four exploratory sets were further assembled into one dataset designated “UIDB” (*k*: 39). Note that this “assembly of assemblies” may contain some misassembled contigs. The UIDB contigs were used as queries to search all insect sequences deposited at GenBank (http://ncbi.nlm.nih.gov/) and *An. stephensi* EST sequences [17] using BLASTX and BLASTN at a cutoff E-value of 1×10^−5^. For discovering process-related genes by quantifying their mRNA level changes, numbers of the UD, ID, UB, and IB reads assembled into each UIDB contig were extracted from the Velvet output and tabulated using Microsoft Excel.

### Read Normalization and Ratio Calculation

Read normalization and ratio calculation were performed as described by Zhang et al [18]. Briefly, based on frequencies of commonly used standards in each of the four libraries (*e.g*. number of rpS3 reads in UD ÷ number of total reads in UD), a set of six ribosomal protein genes were selected as internal standards, which had high total read numbers (>10,000) and low coefficients of variation (*i.e*. SD/mean <10%) in their frequencies. The sums of their read numbers for specific libraries, or library normalization factors (LNFs), which already reflected the differences in library sizes, were directly used to calibrate other read numbers in the corresponding libraries. For a specific contig in UIDB, its relative abundance (RA) in libraries X and Y is defined as: RA_x/y_ = (actual read # in library X ÷ LNF_x_)/(actual read # in library Y ÷ LNF_y_). In case read # in library Y is zero, adjusted read number (ARN) is calculated as: ARN_x_ = actual read # in library X × LNF_y_/LNF_x_. Some of the contigs in UIDB, whose RAs are above a threshold, are categorized as infection induced (UPi: RA_ID/UD_ or RA_IB/UB_ >2), infection suppressed (DNi: RA_ID/UD_ or RA_IB/UB_ <0.5), nitroquine induced (UPn: RA_ID/IB_ or RA_UD/UB_ >2), and nitroquine suppressed (DNn: RA_ID/IB_ or RA_UD/UB_ <0.5). These contigs were used as queries to search insect sequences including *An. stephensi* ESTs, as described in *Section 2.4*.

### Quantitative Real-time PCR Analysis

Quantitative real-time PCR was performed to confirm the RNA-Seq expression profiling by using the same total RNA samples. Eight immunity-related genes were selected for validation of the expression data, including TEP1, APL1C, PGRP, FBN8, Eater, C-type lectin, CLIPC7, and SP (Fig. S1). Two cDNA samples from a similar experiment were examined by quantitative real-time PCR to further verify the RNA-Seq data. In that experiment, the female mosquitoes were fed on *P. yoelii*-infected mice or uninfected mice for 2 h and collected for RNA isolation at 24 h after blood feeding. Specific primers and SYBR Premix EX Taq (TaKaRa, Japan) was used in real-time PCR analysis on an ECO™ Real-Time PCR System (Illumina, USA). The reaction mixture (15 µl total volume) contained 7.5 µl 2×reaction buffer, 0.45 µl primers, 6.05 µl ddH_2_O and 1 µl cDNA template. The ribosomal protein S7 (rpS7) mRNA (GenBank AF539918) was used as an internal control for data normalization with the forward and reverse primers (Fig. S1).

## Results and Discussion

### 1. Identification of Differentially Regulated Genes

To study the effect of *P. yoelii* infection (I) and nitroquine (D for drug) on *An. stephensi* gene transcription, we isolated mRNAs from adult female mosquitoes in the treatment (I and D) and control (U for uninfected, B for buffer) groups. Using oligo-dT primer annealing to the 3′ end of mRNA molecules, we generated four cDNA libraries: UD, ID, UB, and IB. To increase the read number and sequence coverage, we sequenced the libraries on an Illumina GA-IIx HiSeq2000. After removing ∼2% of the reads flagged as low quality or low complexity, we obtained a total of 25,505,495 reads from UD, 25,582,317 from ID, 22,561,525 from UB, and 21,145,138 from IB (Table 1). We established ‘exploratory’ assemblies of the paired end reads in each library at three hash lengths.

**Table 1:** Summary statistics for sequencing analysis of *An. stephensi* ESTs.

		UD	ID	UB	IB
Total number of reads		25,505,495	25,582,317	22,561,525	21,145,138
de novo assembly (Velvet)	*k* = 29	59,959	68,510	44,454	52,096
	*k* = 35	51,626	56,699	45,521	45,552
	*k* = 41	42,329	43,994	37,261	40,819
	Total number of contigs	153,914	169,203	127,236	138,467
*k*: hash length					
contig size (avg./longest in bp)		630/18,654	567/12,990	705/14,638	665/16,526
Total BLASTable sequences		52,813	59,731	41,121	46,350

To quantify changes in gene expression, we also assembled reads from all the four groups into one set of contigs (UIDB) (Table 2). First pass Velvet assemblies with hash length of 21 and 35 yielded 183,137 (N_50_: 231 bp, average size: 209 bp) and 206,003 (N_50_: 271 bp, average size: 230 bp) contigs, respectively. N_50_ value indicates that 50% of the contigs are equal to or longer than this length. Higher hash values of 49 and 59 yielded better assemblies with 85,946 contigs (N_50_: 752 bp, average size: 434 bp) and 48,422 contigs (N_50_: 1,275 bp, average size: 593 bp), respectively. Then we assembled the contigs obtained from these assemblies in a final summary assembly (*k*: 39) and resulted in a final dataset of 32,648 contigs (N_50_: 1819 bp, average size: 1,078 bp), and we have submitted the assembly of UIDB contigs to NCBI Sequence Read Archive database (http://www.ncbi.nlm.nih.gov/Traces/sra_sub/sub.cgi?subid=180492&from=list&action=show: submission) according to Submission Quick Start Guide. The BioProject, BioSample, and Experiment accession numbers are SRP029877, SRS478653 and SRX347875, respectively.

**Table 2:** Summary iterative Velvet assembly UIDB.

	contig number	total length (bp)	average size	N_50_ (bp)	total reads	unused reads
*k* = 21	183,137	38,236,818	208.8	231	188,114,040	145,891,398
*k* = 35	206,003	47,452,332	230.3	271		120,165,694
*k* = 49	85,946	37,272,482	433.7	752		75,851,826
*k* = 59	48,422	28,731,731	593.4	1,275		66,483,158
*k* = 39	32,648	35,182,144	1077.6	1,819	523,508	181,738

We extracted numbers of UD, ID, UB, and IB reads assembled into each UIDB contig. As read numbers directly correlated with library sizes and, therefore, had to be normalized first against control genes. Examination of the frequencies of commonly used internal standards in each of the four libraries resulted in a short list of six genes with low coefficients of variation (<10%) and high total read numbers (>10,000). We used the sums of their read numbers as library normalization factors (LNFs) to calibrate read numbers and calculate relative abundances (RAs). Based on the RA values, 3,046 or 9% of the 32,648 contigs in UIDB were categorized into four groups: infection induced, infection suppressed, nitroquine induced, and nitroquine suppressed. The total reads for each of the 3,046 contigs is greater than 1,000, so that low abundant transcripts (and, therefore, less reliable RA values) are eliminated from our study. To validate the RAs, we performed quantitative real-time PCR on eight genes using the same total RNA samples and found the fold changes were in good agreement in all these groups (Fig. S1). Similar results were obtained using cDNA samples from a different experiment. In the nitroquine induced and suppressed groups, we have found a total of 848 contigs. Among them, 550 contigs (65%) do not have BLAST hits and 111 are related to immunity, oxidative stress, detoxification, cytoskeleton assembling, or cell adhesion. In comparison, there are 2,198 contigs in the infection induced and suppressed groups: 1,806 (82%) have no BLAST hit and 109 are related to the above five processes. The low hit rates may stem from the cDNA synthesis and sequencing methods used.

The Illumina sequencing technology greatly increases read numbers and hence accuracy of RA values, but this comes at a cost. Short read length complicates their assembling into contigs with long coding regions that are highly desirable for species lacking sequenced genomes. Using random primers should alleviate this problem to certain extent, since they favorably bind to GC-rich coding sequences. In contrary, the oligo-(dT) primer causes a bias for contigs containing 3′ untranslated region, which contributes to the low BLAST hit rates of 18–35%. When the *An. stephensi* genome is published, we will overlay all our contigs to the gene models and expand the transcriptome search not only to the groups studied here but also to other processes affected by the drug or infection.

### 2. Sequence Analysis and Function Prediction of Nitroquine-induced Genes

We discovered 356 UIDB contigs whose RA_ID/IB_ or RA_UD/UB_ values were higher than 2. As anticipated, some of these contigs encoded polypeptides either similar to defense proteins identified in *An. gambiae*, *Aedes aegypti*, *Culex quinquefasciatus* and other insects (*e.g*. *Drosophila melanogaster*) (Table 3), or related to proteins previously not known to participate in immune responses, or having no significant sequence similarity to known proteins. In the following sections, we describe them in the order of their putative functions.

**Table 3:** A list of 51 nitroquine-induced contigs for pathogen recognition, signal transduction, oxidative stress, detoxification, cytoskeleton, and cell adhesion proteins.

CIFH contig	Original read#					RA_ID/IB_	RA_UD/UB_	BLAST results
	UD	ID	UB	IB	Total			
**Pathogen recognition**								
26579	238	18	15	8	***279***	2.25	15.87	gb|ADZ44774.1| APL1C [Ag]
25888	100	76	19	35	***230***	2.17	5.26	gb|EAA09703.2| SR CD36 [Ag]
38607	2929	1478	1390	551	6348	2.68	2.11	gb|EAU75744.2| LRR pr. [Ag]
695	2356	1727	859	1150	6092	1.50	2.74	gb|EAL41884.2| LRR pr. [Ag]
51750	1916	1904	944	1782	6546	1.07	2.03	gb|EAA10783.4| LRR pr. [Ag]
12975	1459	2039	580	707	4785	2.88	2.52	gb|ACG68523.1| TEP1 [Ag]
**Extracellular and intracellular signal transduction**								
38768	6544	5141	2952	3024	17661	1.70	2.22	emb|CAA93818.1| SP [Ag]
19862	2057	1178	810	824	4869	1.43	2.54	gb|EAA01781.5| carboxypeptidase [Ag]
37858	79	44	4	30	***157***	1.47	19.75	gb|EAA00311.5| SP [Ag]
13513	57	9	6	28	***100***	0.32	9.5	gb|EDS27643.1| SP rhomboid [Cq]
3479	4949	2874	2261	1304	11388	2.20	2.19	gb|EAA14916.1| SP inhibitor [Ag]
35090	3823	3549	1888	2235	11495	1.59	2.02	gb|EDS26829.1| SP [Cq]
22997	1036	720	394	338	2488	2.13	2.63	gb|AAC02700.1| SP [Ag]
12130	948	1271	367	421	3007	3.02	2.58	emb|CAA80512.1| SP [Ag]
2706	301	2168	1069	170	3708	9.34	0.29	gb|EAA08853.4| clip SP C7 [Ag]
16368	998	725	463	650	2836	1.12	2.13	gb|EAA14062.4| ANK repeat pr. [Ag]
11526	342	67	67	43	519	1.56	5.10	gb|EAA05854.4| ANK repeat pr. [Ag]
3861	106	43	18	56	***223***	0.77	5.89	gb|ABD83643.1| G-nucleotide fact. [Dm]
12518	5	58	73	0	***136***	42.49*	0.09	gb|EAA00858.4| G protein β2 [Ag]
4877	16	34	49	4	***103***	6.23	0.33	gb|AAF52759.2| G protein γ30A [Dm]
16565	1274	802	637	866	3579	0.93	2.00	gb|EAA09222.5| spectrin [Ag]
517	130	835	1525	31	2521	19.73	0.09	gb|EAA10755.2| arrestin 2 protein [Ag]
3493	76	373	1002	16	1467	17.08	0.08	gb|AAG54081.1| arrestin [Ag]
**Oxidative stress and detoxification**								
57082	1825	852	669	285	3631	2.99	2.73	gb|EDS38768.1| sorbitol dehydrogenase [Cq]
12429	0	164	0	17	***181***	7.06	–	gb|ABK97426.1| 14-3-3 epsilon pr. [Bm]
30351	137	5	12	3	***157***	1.67	11.42	gb|EDS33498.1| cytochrome P450 [Cq]
10946	5719	4859	2504	4616	17698	1.05	2.28	gb|EAT44585.1| cytochrome P450 [Aa]
24943	2606	1402	1150	709	5867	2.00	2.27	gb|EAA15034.4| cytochrome P450 [Ag]
**Cytoskeleton and cell adhesion**								
6349	18	76	17	9	***120***	6.18	1.08	gb|EDO63457.1| TGF-β-induced pr. [Ag]
7935	43	36	18	5	***102***	5.28	2.44	gb|EAA00824.1| nexin [Ag]
16374	56	26	5	35	***122***	0.74	11.2	gb|ACO90373.1| myosin H-chain [Bm]
33424	47	40	6	17	***110***	2.35	7.83	ref|XP_970252.2| formin 3 [Dw]
5709	359	209	52	164	784	1.27	6.9	gb|EFN64027.1| myosin L-chain kinase [Ag]
21446	35	34	7	34	***110***	1.03	5.10	gb|EFN83270.1| myosin L-chain kinase [Ag]
16802	9136	6160	4221	5875	25392	1.05	2.16	gb|EAT42759.1| myosin H-chain [Ag]
484	9150	6315	3661	5270	24396	1.2	2.5	gb|EAT46230.1| titin [Aa]
39386	5590	3493	1758	3235	14076	1.08	3.18	gb|EAT42858.1| myosin H-chain [Ag]
722	5514	3851	1642	3053	14060	1.26	3.36	gb|EAT43208.1| myosin L-chain kinase [Aa]
6836	3941	2753	1950	2339	10983	1.18	2.02	gb|EAT36995.1| paramyosin, long [Aa]
19562	1886	1183	628	1210	4907	0.98	3.06	gb|EAT42758.1| myosin H-chain [Ag]
39406	1322	824	459	820	3425	1.00	2.88	gb|EAT42758.1| myosin H-chain [Ag]
49526	1383	513	377	650	2923	0.79	3.67	gb|EAT42758.1| myosin H-chain [Ag]
48089	1225	802	395	778	3200	1.03	3.10	gb|EDS44962.1| myosin H-chain [Cq]
16375	1160	782	368	694	3004	1.13	3.15	gb|EAT42758.1| myosin H-chain [Ag]
14623	863	794	404	747	2808	1.06	2.14	gb|EAT43213.1| titin (connectin) [Aa]
2492	820	579	398	441	2238	1.31	2.06	gb|EAT39116.1| gliotactin [Aa]
14852	953	484	210	470	2117	1.03	4.54	gb|EEB13142.1| titin [Phc]
5828	52	34	8	22	***116***	1.55	6.50	gb|EAT48765.1| cadherin [Aa]
2452	10325	6671	4525	7264	28785	0.94	2.28	gb|EAT45935.1| kakapo [Aa]
302	1132	527	553	802	3014	0.66	2.05	emb|CAA09870.1| kakapo [Dm]

The total read numbers, if lower than 300, are shown in italic and bold so that their RAs need to be interpreted with caution. *:adjusted read number (ARN). The footnote also applies for Tables 4–6.

#### 2.1. Pattern Recognition Receptors (PRRs)

Immune reactions are initiated when microbial surface molecules are recognized as “non-self” by PRRs that bind to pathogen-associated molecular patterns. For example, we found contig 26579 (RA_ID/IB_: 2.25, RA_UD/UB_: 15.87) encode a leucine-rich repeat (LRR) protein homologous to APL1C (Anopheles Plasmodium-responsive LRR protein-1C). There are three other LRR protein contigs (51750, 38607, 695) and one thioester-containing protein (TEP) contig (12975: RA_ID/IB_: 2.88, RA_UD/UB_: 2.52). An. gambiae encodes 24 members of the LRR protein family named LRIMs (for leucine-rich repeat immune proteins). Two of them, LRIM1 and APL1C, are components of a mosquito complement-like system crucial for defense against Plasmodium parasites [22]. LRIM1 and APL1C circulate in the hemolymph exclusively as a disulfide-bonded complex that specifically interacts with the mature form of a complement C3-like protein, TEP1 [23]–[24]. Other recognition proteins include scavenger receptors (SRs, contig 25888, RA_ID/IB_: 2.17, RA_UD/UB_: 5.26). Comparative genomics studies have revealed four orthologs of this receptor in An. gambiae. Knockdown of Croquemort SCRBQ2 expression by dsRNA injection resulted in a 62.5% reduction in oocyst formation, suggesting that this SR plays a key role in Plasmodium-mosquito interactions [25]–[26].

#### 2.2. Extracellular signal modulators

Recognition of non-self usually activates an extracellular cascade of serine proteases (SPs) that amplifies the signal and triggers downstream responses to kill the invaders [27]. We found six SP contigs induced by nitroquine: contigs 38768, 37858, 13513, 35090, 22997, and 12310. SPs share a catalytic mechanism and structural characteristics including three conserved catalytic residues (His, Asp, Ser). Genome annotation of SPs and noncatalytic SPHs (H for homolog) in An. gambiae, D. melanogaster, Homo sapiens, and Fugu rubripes have identified 305, 206, 110, and 125 such genes, respectively [28]. Reverse genetic analyses of several SPs/SPHs demonstrated their importance in mediating melanization, killing, and disposal of malaria parasites in An. gambiae [29]–[31].

Key components of the protease cascades include clip-domain SPs/SPHs (CLIPs), which take part in several defense mechanisms in insects and crustaceans such as the activation of signaling pathways leading to the synthesis of antimicrobial peptides [32], hemolymph coagulation [33], and melanization [34]. We identified a nitroquine-induced homolog of *A. gambiae* CLIPC7 (contig 2706). So far, *An. gambiae* CLIPB14 and CLIPB15 are found to be responsive to bacterial or *Plasmodium* infection: B14 showed persistent up-regulation in *Plasmodium-*infected mosquitoes while B15 showed transient up-regulation during midgut invasion [35]–[36]. Signal transduction mediated by extracellular SP pathways is down regulated by serpins, which are irreversible, suicide inhibitors that covalently bind to the active site Ser of their target SPs [37]. We have identified one nitroquine-induced serpin (contig 3479).

#### 2.3. Intracellular signal transducers

Intracellular immune signaling pathways transmit the alarm signal originated from pathogen- associated PRRs to effector genes. We found two ankyrin repeat protein (ANK) contigs (16368, 11526). Ankyrin repeat is a 33-residue motif often occurring in tandem arrays that cooperatively fold into structures for molecular recognition via protein interactions. *An. gambiae* REL2 (a Relish homolog containing an ANK domain) regulates expression of antimicrobial peptide genes cec1, cec3, and gam1 [38]. Spectrins are components of G-protein coupled receptor (GPCR) and synaptic multi-protein complexes involved in cell cycle by regulating expression of membrane receptors [39]. Invertebrates have a small repertoire of spectrin genes. We found aspectrin (contig 16565), G-protein components (contigs 12518 and 4877), as well as arrestins (contigs 517 and 3493). Arrestins mediate cellular processes via interactions with secondary signal transduction cascades by means of recruiting and activating mitogen-activated protein kinase (MAPK) and other effectors [40]–[41]. Other intracellular proteins possibly involved in signal transduction or modulation include a GTP/GDP exchange factor.

#### 2.4. Oxidative stress and detoxification proteins

Several genes for oxidative stress and detoxification were up-regulated by nitroquine, including sorbitol dehydrogenase (contig 57082, RA_ID/IB_: 2.99, RA_ UD/UB_: 2.73), 14-3-3 epsilon protein (contig 12429), three cytochrome P450 contigs (30351, 10946, 24943).

#### 2.5. Cytoskeleton and cell adhesion molecules

Cytoskeletal dynamics and remodeling are considered as key factors affecting P. berghei invasion of the mosquito midgut [12]. Nitroquine affected the expression of some cytoskeletal and adhesion proteins. A highly conserved, transforming growth factor (TGF) -β inducible matrix protein (contig 6349, RA_ID/IB_: 6.18) may be a bifunctional linker between individual matrix components and resident cells [42]. Six innexin genes in *Ae. aegypti* are closely related to their *Drosophila* homologs, suggesting critical roles of gap junctions in diverse cellular and tissue functions [43]. We identified an innexin gene (contig 7935, RA_ID/IB_: 5.28, RA_ UD/UB_: 2.44) in An. stephensi, which was highly induced after the drug treatment. A putative formin (contig 33424, RA_ID/IB_: 2.35, RA_ UD/UB_: 7.83) may govern microtubule and microfilament dynamics by functioning as an effector of ρ small GTP-binding proteins during cell adhesion, cytokinesis, polarization, and morphogenesis [44]. Titin (contigs 484, 14623, and 14852), the third most abundant protein in muscle (after actin and myosin), is responsible for much of the myofibril elasticity in various organisms. Titin molecules are associated with each thick filament in skeletal muscle, where they interact with myosin [45]. Ten myosin contigs were up-regulated (Table 3). Gliotactin (contig 2492), first identified in the tricellular junction, is necessary for the development of tricellular junction and septate junctions [46]. Cadherin (contig 5828, RA_ UD/UB_: 6.50) is a cell surface glycoprotein that may act as a receptor for envelop proteins of dengue or West Nile viruses when they enter mosquito cells [47]. Two kakapo protein contigs (2452, 302) encode a giant cytoskeleton protein that has multiple isoforms with characteristics of the spectrin and plakin superfamilies. Previously characterized short isoforms are similar to spectrin and dystrophin with an actin-binding domain followed by spectrin repeats [48].

### 3. Nitroquine-suppressed Genes

We further analyzed the 492 contigs down-regulated by nitroquine. Among the 158 (32%) with BLAST hits, 60 contigs were related to defense responses (Table 4). These include two PGRP contigs (42111, RA_UD/UB_: 0.12; 42110, RA_UD/UB_: 0.16). PGRPs are soluble or membrane- bound proteins with a domain similar to T7 lysozyme that hydrolyzes bacterial peptidoglycans 4-[49]. We also found three fibrinogen-related protein (FREP) contigs (54250, 9243, 6608). FREPs belong to an evolutionarily conserved gene family in mammals and invertebrates. *An. gambiae* has as many as 59 FREPs whereas *D. melanogaster* only has 14 [50]. A majority of these genes display immune-responsiveness after challenge with bacteria, fungi or *Plasmodium,* and their expression patterns correlate strongly with gene phylogeny and chromosomal location. We identified two putative Eater contigs (9561, 4173). In the fruit fly, this SR with epidermal growth factor-like repeats is produced by phagocytes and required to survive after Gram-positive or -negative bacterial infection [51]. Among the nitroquine-suppressed genes, there are eight prophenoloxidase (PPO) contigs (13019, 7548, 18477, 24062, 24063, 3605, 3163, 29061) (Table 4). It is generally accepted that insect phenoloxidases (POs) are synthesized as inactive PPOs that undergo limited proteolysis by specific SPs to become active. Insect POs are key enzymes causing melanization around invading pathogens and at wounds to prevent infection [52]. We also found six SP contigs (700, 13660, 10250, 47533, 20619, 47687) and one defensin contig (22126, RA_UD/UB_: 0.20). Additionally, we identified ten down-regulated genes related to oxidative stress and detoxification: two NADH dehydrogenase contigs 19543 and 21976, five cytochrome oxidase contigs (26093, 37377, 12844, 12660, 37606), peroxidase contig 38629, esterase A11 contig 49194, and carboxylesterase contig 2792.

**Table 4:** A list of 60 nitroquine-suppressed contigs for pathogen recognition, signal transduction, effector, oxidative stress, detoxification, cytoskeleton, and cell adhesion proteins.

UIDB contig	Original read#					RA_ID/IB_	RA_UD/UB_	Blast results
	UD	ID	UB	IB	Total			
**Pathogen recognition**								
9561	56	88	42	132	318	0.49	1.37	gb|ADK20118.1| Eater [Dm]
4173	150	93	71	139	453	0.49	2.17	gb|ADK20124.1| Eater [Dm]
42111	33	95	287	94	509	0.74	0.12	gb|ADA54914.1| PGRP [Aa]
42110	126	505	785	475	1891	0.78	0.16	gb|ACI30152.1| PGRP [Ad]
54250	7	45	32	23	***107***	1.45	0.22	gb|ADC29801.1| FBN8 [Ag]
6608	35	136	76	164	411	0.61	0.47	gb|EAT36947.1| fibrinogen/fibronectin [Aa]
9243	192	230	112	448	982	0.38	1.75	gb|ADF80532.1| FBN9 [Ag]
1559	35	24	41	36	***136***	0.87	0.49	gb|EFN79581.1| SR-B1 [Hs]
**Extracellular and intracellular signal transduction**								
700	166	181	82	300	729	0.44	2.08	gb|EAT40468.1| SP [Aa]
13660	9	43	23	34	***109***	0.40	0.93	gb|EDS40340.1| SP [Cq]
10250	227	635	580	339	1781	1.39	0.40	gb|EAT33372.1| SP [Aa
47533	251	569	570	199	1589	2.08	0.45	gb|ACN38211.1| SP14 [Ag]
20619	1506	1721	960	2533	6720	0.50	1.61	gb|EDS32243.1| SP nudel[Cq]
47687	36	186	83	46	351	2.94	0.44	gb|ACN38197.1| SP14 [Ag]
4825	31	17	32	33	***113***	0.38	1.00	gb|EAT43503.1| MRAS2 [Aa]
32102	55	28	39	52	***174***	0.40	1.45	gb|EAT33735.1| G nucleotide-BP β3
25178	40	43	15	67	***165***	0.47	2.78	gb|AAF53349.2| centaurinγ1A [Dm]
28679	412	205	286	319	1222	0.47	1.47	gb|EAT42068.1| microtubule kinase [Aa]
8919	41	23	6	34	***104***	0.50	7.69	gb|EAT37086.1| netrin receptor (unc5) [Aa]
31670	14	49	198	34	***295***	1.06	0.07	gb|AAV84215.1| EF1α [Cs]
698	65408	69153	155352	79238	369151	0.64	0.43	gb|EDS27326.1| GH regulated TBC pr1 [Cq]
2295	22	69	46	29	***166***	1.75	0.49	gb|EDS27643.1| rhomboid [Cq]
46422	66	24	24	59	***173***	0.30	2.86	gb|EDS25690.1| GTP-BPα, gnao [Cq]
**Effectors**								
22126	1986	6575	9960	4674	23195	0.97	0.20	gb|ABB00946.1| defensin [Ag]
13019	18	29	38	49	***134***	0.43	0.49	gb|AAC69182.1| PPO [As]
7548	96	253	124	598	1071	0.31	0.81	gb|AAB94672.1| PPO3 [Ag]
18477	56	106	58	247	467	0.31	0.99	gb|AAB94672.1| PPO3 [Ag]
24062	68	181	91	401	741	0.33	0.76	gb|AAB94672.1| PPO3 [Ag]
24063	114	254	184	521	1073	0.36	0.63	gb|AAB94672.1| PPO3 [Ag]
3605	41	79	48	162	330	0.36	0.88	gb|AAB94672.1| PPO2 [Ag]
3163	877	1681	1034	3172	6764	0.39	0.87	gb|EAA10430.2| PPO9 [Ag]
29061	117	121	53	217	508	0.41	2.27	gb|AAD01936.1| PPO [Ag]
**Oxidase stress and detoxification**								
19543	43	11	26	102	***182***	0.11	1.20	gb|ADE18217.1| NADH DH-4 [Bm]
26093	54	20	35	124	***233***	0.17	1.14	gb|ACG59301.1| cytochr. C oxidase-3 [Gp]
37377	132	28	82	149	391	0.19	1.18	gb|ABV71224.1| cytochr. oxidase-2 [Dt]
21976	15	12	26	61	***114***	0.20	0.42	gb|ADO60622.1| NADH DH-1 [Of]
12844	44	18	28	61	***151***	0.30	1.15	gb|ABH03691.1| cytochr. b [Cd]
12660	190	88	141	223	642	0.40	0.99	gb|AAT69281.1| cytochr. c oxidase-1 [Td]
37606	1129	411	1241	991	3772	0.42	0.67	gb|ABF18100.1| cytochr. c oxidase[Aa]
38629	57	24	66	51	***198***	0.48	0.63	gb|AAV30070.1| peroxidase 4A [Ag]
49194	46	20	36	40	***142***	0.37	1.32	gb|ABO85014.1| esterase A11 [Cq]
2792	313	288	195	474	1270	0.45	1.64	gb|EDS29180.1| carboxylesterase 3 [Cq]
**Cytoskeleton and cell adhesion**								
4610	57	14	22	43	***136***	0.24	2.70	gb|EDS37841.1| kakapo [Cq]
17301	72	14	28	41	***155***	0.25	2.63	gb|EFN61557.1| NCAM2 [Cf]
27643	78	11	17	31	***137***	0.26	4.76	gb|AAN10358.5| dumpy [Dm]
52735	33	18	18	38	***107***	0.35	1.89	gb|EAT36301.1| perlecan [Aa]
2620	100	54	119	98	371	0.40	0.86	gb|EDS31391.1| cadherin [Cq]
24227	153	42	31	67	***293***	0.46	5.26	gb|EDS41643.1| dumpy [Cq]
44130	160	124	75	184	543	0.50	2.17	gb|EAT36301.1| perlecan [Aa]
37846	63	30	29	44	***166***	0.50	2.22	gb|EAT48357.1| cadherin [Aa]
35484	27	20	18	46	***111***	0.32	1.54	gb|EAT42208.1| paraflagellar rod pr. [Aa]
8549	55	25	35	52	***167***	0.35	1.61	gb|EDS28887.1| myosin III [Cq]
11346	62	57	41	106	***266***	0.40	1.54	gb|EDS31801.1| rab6 activating pr. [Cq]
6175	50	26	39	39	***154***	0.49	1.32	gb|AAX52757.1| formin 3A [Dm]
46683	12	59	62	60	***193***	0.72	0.20	gb|EDS37500.1| actin [Cq]
18610	24	119	109	62	314	1.41	0.23	gb|EAT38677.1| actin [Aa]
24214	26	59	58	52	***195***	0.83	0.46	gb|EDS37500.1| actin [Cq]
10559	24	21	50	21	***116***	0.74	0.49	gb|AAV65298.1| actin [Ag]
36525	45	74	183	67	369	0.81	0.25	gb|EAT34065.1| actin BP [Aa]
5097	53	13	79	31	***176***	0.68	0.31	gb|EFN75894.1| whirlin [Hs]

In the previous study [13], at five days post-feeding on *P. yoelii* infected mice, mosquitoes were continually fed on 0.1% nitroquine in sucrose solution prior to collection at 7, 9, and 13 days post infection for morphology, PCR, and PO activity tests. Under those conditions, we observed melanized oocysts and an increase in PPO mRNA levels in the nitroquine-treated mosquitoes. Here, we administered the drug to *P. yoelii*-infected mice at a dosage similar to that for people in malaria endemic areas, but did not observe melanized ookinetes and detected a decrease in PPO mRNA levels. A likely reason for the discrepancy is the low dosage and no drug administration after the blood meal, which caused a milder immune response that hampered but did not completely block the parasite development. There was no clear difference in gametocyte number or morphology between the ID and IB groups after treatment (data not shown). Nonetheless, we detected significant decrease in median oocyst count at 10 days post- blood feeding (Fig. S2), indicating that pre-oocyst stages (including those in mouse) were affected by low dosage of nitroquine but stayed viable for a period of time. After all, nitroquine did cause complex changes in gene expression.

### 4. Genes Induced by Plasmodium Infection

#### 4.1. Pattern recognition receptors

While mRNA levels are up- or down- regulated after nitroquine treatment, we also examined how parasite infection may affect transcription (Tables 5 and 6) and identified overlaps with the contigs in Tables 3 and 4. Two FREP contigs (54250, RA_ ID/UD_: 5.99; 9243, RA_ IB/UB_: 5.20) and one Eater (contig 9561) were up-regulated in infected mosquitoes but down-regulated in drug-treated ones. We found one C-type lectin (contig 4534), one complement 4b-like protein (contig 4650), and one TEP (contig 13138). The A. gambiae genome contains 19 TEP genes [49]. TEP1 is a typical one characterized in some detail [53]. It is unclear whether all mosquito TEPs are engaged in similar functions. An. gambiae *TEP3* and *TEP4* are up-regulated upon bacterial challenge or parasite infection [54]. Two LRR protein contigs (2407 and 4502) had RA_ IB/UB_ of 2.07 and 2.38, respectively.

**Table 5:** A list of 89 infection-induced contigs for pathogen recognition, signal transduction, effector, oxidative stress, detoxification, cytoskeleton, and cell adhesion proteins.

UIDB contig	Original read#								RA_ID/UD_		RA_IB/UB_	Blast results
	UD	ID				UB	IB	Total				
**Pathogen recognition**												
54250	7		45			32	23	***107***	5.99	0.93		gb|EAA00222.4| FBN8 [Ag]
9243	192		230			112	448	982	1.11	5.20		gb|AAR13732.1| FBN [Ag]
9561	56		88			42	132	318	1.46	4.08		gb|ADK20118.1| Eater [Dm]
13138	61		59			22	37	***179***	0.90	2.18		gb|AAM52596.1| TEP2 [Dm]
4650	505		458			272	526	1761	0.85	2.51		gb|EDS39639.1| c4b-binding protein [Cq]
7569	555		462			272	523	1812	0.77	2.50		gb|ADK20110.1| Eater [Dm]
4534	221		265			191	298	975	1.11	2.03		gb|EAA08886.5| C-type lectin [Ag]
2407	253		500			177	282	1212	1.84	2.07		gb|EAT46201.1| LRR [Aa]
4502	208		281			158	290	937	1.26	2.38		gb|EDS45577.1| LRR [Cq]
**Extracellular and intracellular signal transduction**												
479	382			1216		650	1442	3690	2.96	2.89		gb|EAT45655.1| SP [Aa]
2792	313			288		195	474	1270	0.86	3.15		gb|EDS29180.1| carboxylesterase 3 [Cq]
20619	1506			1721		960	2533	6720	1.07	3.43		gb|EDS32243.1| SP [Cq]
2323	960			1006		581	1148	3695	0.98	2.57		gb|EDS32243.1| SP[Cq]
41006	799			903		507	806	3015	1.05	2.07		gb|AAS99341.1| carboxypeptidase B [Ag]
14021	722			1425		369	870	3386	1.84	3.06		gb|EDS33630.1| carboxypeptidase A1 [Cq]
17844	0				109	0	30	***139***	101.59	38.98		gb|EDW63808.1| G nucleotide-BP [Dv]
3643	123				427	67	604	1221	3.23	11.71		gb|AAF48160.3| protein kinase Cδ-C [Dm]
8154	477				446	218	383	1524	0.87	2.28		gb|EAA14062.4| ANK pr. [Ag]
13991	617				984	487	1158	3246	1.49	3.09		gb|AAF49192.2| MAPK[Dm]
18201	383				693	303	543	1922	1.69	2.33		gb|EDS30611.1| caspase-2 [Cq]
4898	315				367	184	295	1161	1.09	2.08		gb|EAT33004.1| ankyrin [Aa]
21463	348				299	145	331	1123	0.80	2.96		gb|EDW32992.1| GPCR [Dp]
**Effectors**												
22126	1986				6575	9960	4674	23195	3.09	0.60		gb|ABM92299.1| defensin [As]
7548	96				253	124	598	1071	2.45	6.26		gb|AAC69182.1| PPO [As]
10561	365				399	309	583	1656	1.02	2.45		gb|EDS44938.1| PO1 [Cq]
24062	68				181	91	401	741	2.48	5.72		gb|AAB94672.1| PPO2 [Ag]
18477	56				106	58	247	467	1.76	5.53		gb|AAO22166.1|PPO [Ac]
29061	117				121	53	217	508	0.96	5.31		gb|AAD01936.1| PPO [Ag]
24063	114				254	184	521	1073	2.08	3.67		gb|AAB94672.1| PPO2 [Ag]
39231	72				128	76	169	445	1.67	2.88		gb|AF062034.1| PPO [As]
3605	41				79	48	162	330	1.80	4.38		gb| AAF57775.1| PPO-A1 [Dm]
3163	877				1681	1034	3172	6764	1.78	3.98		gb|EAA10430.2| PPO9 [Ag]
**Oxidative stress and detoxification**												
37606	411				1129	991	1241	3772	2.56	1.62		gb|ABF18100.1| cytochr. c oxidase [Aa]
10946	5719				4859	2504	4616	17698	0.79	2.39		gb|EAT44585.1| cytochr. P450 [Aa]
12511	351				428	181	382	1342	1.13	2.74		gb|EAT40234.1| cytochr. P450 [Aa]
18603	352				581	318	782	2033	1.53	3.19		gb|EAT32533.1| oxidase[Aa]
12532	225				326	152	351	1054	1.35	3.00		gb|EAU77317.2| cytochr. P450[Ag]
12429	0				164	0	17	***181***	152.84	22.09		gb|ABK97426.1| 14-3-3 epsilon[Bm]
4356	1757				2396	1330	2962	8445	1.27	2.89		gb|AAA02748.1| oxidase [Dm]
3114	416				462	329	545	1752	1.03	2.15		gb|EAT46755.1| cytochr.P450 [Aa]
10978	609				496	337	536	1978	0.75	2.07		gb|EDS44734.1| esterase B1 [Cq]
10811	574				610	330	513	2027	0.99	2.01		gb|EAT40232.1| cytochrome P450 [Aa]
14692	545				434	244	387	1610	0.74	2.06		gb|EAT37005.1| cytochrome P450 [Aa]
8206	562				472	203	404	1641	0.78	2.58		gb|EDS31351.1| peroxidase [Cq]
**Cytoskeleton and cell adhesion**												
14827	0				304	0	50	354	283.32*	64.97*		gb|EAA09442.4| microtubule BP [Ag]
5354	0				253	1	99	353	235.79*	128.63		gb|EAT47479.1| myosin H-chain [Ag]
16374	56				26	5	35	***122***	0.43	9.09		gb|EAT42758.1| myosin H-chain [Ag]
39386	5590				3493	1758	3235	14076	0.58	2.39		gb|EAA43613.3| myosin H-chain [Ag]
19562	1886				1183	628	1210	4907	0.58	2.50		gb|EAA43613.3| myosin H-chain [Ag]
3055	180				559	113	725	1577	2.89	8.33		gb|EAT42933.1| myosin L-chain [Aa]
12618	170				442	137	509	1258	2.42	4.82		gb|EDS45745.1| myosin L-chain [Cq]
38163	0				73	0	31	***104***	68.03	40.28		gb|EEB15484.1| paramyosin [Phc]
4689	0				111	0	51	***162***	103.45	66.27		gb|AAA56881.1| actin 1D [Ag]
26092	165				308	109	362	944	1.73	4.31		gb|EAL38580.4| actin-binding activator [Ag]
8662	15				120	19	114	***268***	7.45	7.79		gb|EAT41026.1| stretchin-mlck [Aa]
34885	8				52	2	56	***118***	6.05	36.38		gb|AAF58087.2| stretchin-mlck-D [Dm]
4450	15				109	14	80	***218***	6.77	7.42		gb|AAF58087.2| stretchin-mlck-D [Dm]
21446	35				34	7	34	***110***	0.90	6.31		gb|EAT41026.1| stretchin-mlck [Aa]
7262	67				1856	261	1984	4168	25.82	9.87		gb|EAA10802.5| troponin[Ag]
5116	56				45	13	55	***169***	0.75	5.49		gb|EAT48765.1| cadherin [Aa]
2452	10325				6671	4525	7264	28785	0.60	2.09		gb|EAT45935.1| kakapo [Aa]
2020	2823				4398	3775	7178	18174	1.45	2.47		gb|ACJ10211.1| aminopeptidase N[Ag]
127	3457				3522	2562	4142	13683	0.95	2.10		gb|EDS25698.1| calsyntenin-1 [Cq]
722	5514				3851	1642	3053	14060	0.65	2.42		gb|EAT43208.1| myosin L-chain [Aa]
693	2329				2616	1280	2330	8555	1.05	2.37		gb|EAA07720.5| cadherin [Ag]
2560	2051				1764	1319	2070	7204	0.80	2.03		gb|EDS41979.1| myosin-VIIa [Cq]
1270	2471				1661	808	1373	6313	0.62	2.21		gb|AAS65018.1| mucin [Dm]
25771	576				1014	474	1040	3104	1.64	2.85		gb|EAT41121.1| aminopeptidase [Aa]
4345	973				683	497	909	3062	0.65	2.38		gb|EDW70354.1| cadherin [Dv]
8316	726				827	486	892	2931	1.06	2.38		gb|EAL40462.3| cadherin [Ag]
2453	751				961	488	828	3028	1.19	2.20		gb|EAT45302.1| caspase-1 [Aa]
39406	1322				824	459	820	3425	0.58	2.32		gb|EAA43613.3| myosin H-chain[Ag]
48089	1225				802	395	778	3200	0.61	2.56		gb|EDS44962.1| myosin H-chain [Cq]
35309	503				668	413	734	2318	1.23	2.31		gb|EAT42696.1| aminopeptidase [Aa]
51964	954				606	403	704	2667	0.59	2.27		gb|EDS33424.1| spectrin α-chain [Cq]
16375	1160				782	368	694	3004	0.62	2.45		gb|EDW64148.1| myosin H-chain [Dv]
49526	1383				513	377	650	2923	0.35	2.24		gb|EAA43613.3| myosin H-chain[Ag]
2649	1120				510	291	664	2585	0.42	2.96		gb|AAF58087.2| stretchin-mlck[Dm]
14255	578				495	366	569	2008	0.80	2.02		gb|EAT36338.1| cell adhesion mol. [Aa]
2742	574				450	351	574	1949	0.73	2.12		gb|EAT39367.1| cell adhesion mol. [Aa]
2087	512				581	320	593	2006	1.06	2.41		gb|EAT39611.1| myosin vii [Aa]
14852	953				484	210	470	2117	0.47	2.90		gb|EAT46231.1| myomesin [Aa]
5560	383				362	258	416	1419	0.88	2.10		gb|EDO63657.1| myosin [Ag]
14827	0				304	0	50	354	283.32	64.97		gb|EAA09442.4| microtubule BP [Ag]
5354	0				253	1	99	353	235.79	128.63		gb|EAT47479.1| myosin H-chain [Ag]
16374	56				26	5	35	***122***	0.43	9.09		gb|EAT42758.1| myosin H-chain [Ag]
39386	5590				3493	1758	3235	14076	0.58	2.39		gb|EAA43613.3| myosin H-chain [Ag]
19562	1886				1183	628	1210	4907	0.58	2.50		gb|EAA43613.3| myosin H-chain [Ag]
3055	180				559	113	725	1577	2.89	8.33		gb|EAT42933.1| myosin L-chain [Aa]

**Table 6:** A list of 20 infection-suppressed contigs for immunity, cell adhesion, and oxidative stress proteins.

UIDB contig	Original read#					RA_ID/UD_		RA_IB/UB_	BLAST results
	UD	ID	UB	IB	Total				
**Immunity**									
17359	2472	1219	1320	1639	6650	1.61	0.46		gb|EAL40577.4| ANK repeat pr. [Ag]
11526	342	67	67	43	519	0.18	0.84		gb|EAA05854.4| ANK repeat pr. [Ag]
4877	16	34	49	4	***103***	2.00	0.11		gb|EAA13974.3| G-nucleotide-BPγ [Ag]
12518	5	58	73	0	***136***	11.11	0.00		gb|EAA00858.4| G-nucleotide-BP [Ag]
3493	76	373	1002	16	1467	4.76	0.02		gb|AAG54081.1| arrestin [Ag]
517	130	835	1525	31	2521	6.25	0.03		gb|EAA10755.2| arrestin 2-like protein [Ag]
22758	75	48	56	8	***187***	0.60	0.19		gb|EAT37101.1| SP [Aa]
6751	3235	1414	1659	1356	7664	0.41	1.06		gb|ABE97919.1| SP [Ad]
7395	1324	477	514	197	2512	0.50	0.34		gb|ABF18223.1| hemolymph pr. [Aa]
2706	301	2168	1069	170	3708	6.67	0.21		gb|EAA08853.4| CLIPC7 [Ag]
26579	238	18	15	8	***279***	0.07	0.69		gb|EAA04533.4| LRR protein APL1C [Ag]
38607	2929	1478	1390	551	6348	0.47	0.52		gb|EAU75744.2| LRR transmembr. pr. [Ag]
27840	920	274	313	190	1697	0.28	0.79		gb|AAR05800.1| ICHIT [Ag]
**Cell adhesion**									
302	1132	527	553	802	3014	0.43	1.89		gb|EAA12983.4| kakapo [Ag]
27643	78	11	17	31	***137***	0.13	2.38		gb|AAN10358.5| dumpy [Dm]
20370	107	21	28	43	***199***	0.18	2.00		gb|EAA00978.5| AGAP001632-PA [Ag]
729	3427	1704	932	1608	7671	0.46	2.27		gb|EAT40358.1| calmin [Aa]
7805	1621	350	437	278	2686	0.20	0.83		gb|EAA11173.3| peritrophic matrix pr. [Ag]
13513	57	9	6	28	***100***	0.15	6.25		gb|EDS27643.1| rhomboid [Cq]
**Oxidative stress**									
30351	137	5	12	3	***157***	0.03	0.33		gb|EGK96893.1| cytochrome P450 [Ag]

#### 4.2. Other up-regulated immunity-related genes

Similarly, nine PPO contigs (7548, 10561, 24062, 18477, 29061, 24063, 39231, 3605, 3163) were up-regulated in infected mosquitoes (Table 5). There are over 20 PPOs identified in *Ae. aegypti*, *An. gambiae*, *An. stephensi*, *An. culicifacies* and *Ar. subalbatus*, with multiple PPOs in each species [55]. We also found three SP contigs were induced by parasite infection (contigs 479, 20619, 2323). Several intracellular proteins possibly involved in signal transduction/modulation. These include two GTP/GDP exchange factors, two ANK proteins and one MAPK. One defensin (contig 22126, RA_ID/UD_: 3.09) was up-regulated in P. yoelii-infected mosquitoes, which was down-regulated after nitroquine treatment.

#### 4.3. Oxidative stress and detoxification proteins

Ten oxidative stress-responsive and detoxification genes were up-regulated: 7 cytochrome oxidase contigs (37606, 10946, 12511, 12532, 3114, 10811, 14692), a peroxidase (contig 18603), an oxidase (contig 4356), an esterase (contig 10978) and a 14-3-3 epsilon protein (contig 12429). Reactive oxidative species (ROS) can cause cell damage and, hence, are rapidly inactivated in cells by oxygen scavengers and reduction reactions. The inactivation is facilitated by enzymes such as peroxidases and cytochrome c [56]. It is intriguing that transcription of these enzymes was down-regulated after nitroquine treatment but up-regulated in P. yoelii-infected mosquitoes, indicating that nitroquine may suppress the production of these enzymes. Insufficient reduction of ROS may cause damage in parasite by reacting with its lipids, proteins, and nucleic acids. The imbalance of free radical-generating and -scavenging mechanisms then led to loss of homeostasis and Plasmodium death. The involvement of ROS in mosquito immunity against bacteria and Plasmodium was investigated in the malaria vector An. gambiae [57]. The production of ROS is thought to contribute to the refractory phenotype, as experimentally elevating ROS in susceptible mosquitoes makes them more refractory [58]. Similarly, a variety of synthetic insecticides are known to suppress the activity of key reducing enzymes [59]–[60].

#### 4.4. Cytoskeleton and cell adhesion molecules

Several induced proteins involved in cytoskeleton formation and cell adhesion, including one microtubule binding protein (contig 14827, RA_ID/UD_: 283.32) and seven myosin-related contigs (5354, 16374, 39386, 19562, 3055, 12618, 38163). Myosin was identified as microtubule binding protein by co-sedimentation analysis in the presence of microtubules [61]. Two actin-related protein contigs (4689, 26092) and four stretchin-mlck contigs (8662, 4450, 34885, 21446) were also induced. Members of the titin/myosin light chain kinase family play essential roles in organization of the actin/myosin cytoskeleton, especially in sarcomere assembly and function [62].

### 5. Genes Suppressed by Plasmodium Infection

Interestingly, while certain immunity-related genes were induced by nitroquine treatment, some of them were down-regulated upon *P. yoelii* infection. For instance, APL1C (contig 26579) and LRR protein (contig 38607) had RA_ID/UD_ of 0.07 and 0.47, respectively. CLIPC7 (contig 2706, RA_IB/UB_: 0.21), three SP contigs (7395, 22758, 6751), two ANK contigs (17359, 11526), two arrestin contigs (517, 3493), and two guanine nucleotide-binding protein contigs (4877, 12518) showed similar patterns of gene suppression. In addition, contig 27840 (RA_ID/UD_: 0.28) encodes ICHIT, whose two chitin-binding domains are linked by a Thr-rich linker. The protein, induced by both bacteria and malaria parasite infection [63], may associate with peritrophic matrix, a chitinous sac separating blood meal from midgut epithelial cells.

The high oocyst number in midgut (Fig. S2) and the RAs of infection-affected genes suggest that *P. yoelii* (By265) and *An. stephensi* (Hor) form a highly compatible vector-parasite pair in which the host immune system is no longer effective. Jaramillo-Gutierrez et al. [64] showed that silencing several genes involved in oxidative (OXR1 and GSTT1) or immune (LRIM1 and CTL4) responses had no effect on *P. yoelii* (17XNL) infection of *An. stephensi* (Nijmegen Sda500). In this study, nitroquine induced immune responses of *An. stephensi* (Hor) by unknown mechanism. In nitroquine-treated mosquitoes, mRNA levels of TEP1, APL1C (contig 26579), and other LRR proteins (contigs 38607, 695, and 51750) increased (Table 3). The *Plasmodium* infection induced FREPs, TEP2, and other PRRs (Table 5) but suppressed APL1C (contig 26579) and the LRR protein (contig 38607) (Table 6). While these observations are interesting, we cannot conclude whether this fluctuation of different PRR mRNA levels is responsible for the reduction of *P. yoelii* infectivity in nitroquine-treated mosquitoes. Functional data from RNAi experiments may inform us more on the roles of these PRRs in governing the fate of *P. yoelii* in the mosquito. We are also cautioned by the opposite effects of chloroquine and nitroquine on mosquito response to *Plasmodium* infection, as well as the differences caused by sampling time and method of drug administration. It is desirable to assess whether chloroquine, nitroquine, and other antimalarial drugs have similar effects on the mosquito response at malaria endemic regions.

## Conclusion

Our study shows nitroquine has a significant impact on transcript abundances of certain mosquito genes implicated in defense against *Plasmodium*, which encode pathogen recognition receptors, signal transducers/modulators, and cytoskeleton-, adhesion-, and oxidative stress- related proteins. Such changes may stimulate the innate immune system of *An. stephensi* to fight *P. yoelii* infection. Even though we do not understand the mechanisms of this drug effect, its impact on the mosquito is evident from the present and previous studies. Together, the results suggest nitroquine induces the production of LRIM1/APL1C/TEP1 complex and down- regulation of enzymes involved in ROS reduction, enhances the attack on parasites, and decreases the *Plasmodium* infectivity. Mechanisms for the infectivity decrease may be distinct, when the time or method of nitroquine administration is different.

## Supporting Information

Figure S1 **Confirmation of the RNA-Seq expression profiles by quantitative real-time PCR using the same RNA samples (ID, UD, UB, and IB) (A) and two different RNA samples from a similar test (B).**
(DOC)

Figure S2 **Comparison of oocyst counts in the infected mosquitoes fed on nitroquine- and buffer-treated mice.**
(DOC)
