# Supplementary material for: Modulation of Anopheles stephensi Gene Expression by Nitroquine, an Antimalarial Drug against Plasmodium yoelii Infection in the Mosquito
Source: PLoS One. 2014 Feb 24;9(2):e89473. doi: 10.1371/journal.pone.0089473 (PMC3933544; doi:10.1371/journal.pone.0089473)
Supplement: Figure S1 — Confirmation of the RNA-Seq expression profiles by quantitative real-time PCR using the same RNA samples (ID, UD, UB, and IB) (A) and two different RNA samples from a similar test (B). (DOC) [file pone.0089473.s001.doc]

**A**

**B**

**Figure S1.** Confirmation of the RNA-Seq expression profiles by qRT-PCR using the same RNA samples (ID, UD, UB, and IB) (**A**) and two different RNA samples from a similar test (**B**). In that test (in lieu of UB and IB biological replicates), the female mosquitoes were fed on *P. yoelii*-infected mice or uninfected mice for 2 h and collected at 24 h after blood feeding. Eight immunity-related genes were selected for validating the RA data from RNA-Seq analysis. These genes are thioester-containing protein-1 (TEP1, ACG68523), *Anopheles Plasmodium*-responsive LRR protein-1C (APL1C, ADZ44774), peptidoglycan recognition protein (PGRP, ADA54914), fibrinogen-related protein-8 (FBN8, ADC29801), Eater (ADK20118), C-type lectin (EAA08886), clip-domain serine protease-C7 (CLIPC7, EAA08853), and serine protease (SP, EAT37101). The ribosomal protein S7 (rpS7, AF539918) mRNA was used for normalization. The primer pair and amplicon size are: 5'-ACGACGGCTTCAATAACGAT-3’, 5'-CCCGAGTT CCAGTTCCACTA-3' and 158 bp for TEP1, 5'-CGTGGTAGCGTTCTCCTGAC-3', 5'-GCTGG GACTTCATCACAATC-3' and 172 bp for APL1C, 5'-GCCTCCAACTACTGGCTGAT-3', 5'-AAGACGGACGATGGCACA-3' and 152 bp for PGRP, 5'-CGGCTGGATGTGTTTACCG-3', 5'-CGCCCATTACGATGACTTTAG-3’ and 250 bp for FBN8, 5'-GCTGGTGGCTGTATCGTG TA-3', 5'-AGTTTGCTCCCTGTGATTGC-3' and 164 bp for Eater, 5'-AACGAATCACCACCA TCCAG-3', 5'-CGACCTTCTCCTTTGTCTCG-3' and 150 bp for C-type lectin, 5'-GCAAACAA ACCAGCGTGATA-3', 5'-GGGATACACCGAAGCGATAA-3' and 159 bp for CLIPC7, 5'- AT GGACAGACAGCCAAGAGG-3', 5'-CAAAGCGTGATGTGGGAAT-3' and 197 bp for SP, and 5’-CTAACGACACGAAGACCACAAGA-3’, 5’-CAACCTGCAACGAAGCAAAA -3’ and 154 bp for rpS7. Annealing temperatures of the qRT-PCRs of all transcripts are 60ºC.
