# Supplementary material for: Modulation of Anopheles stephensi Gene Expression by Nitroquine, an Antimalarial Drug against Plasmodium yoelii Infection in the Mosquito
Source: PLoS One. 2014 Feb 24;9(2):e89473. doi: 10.1371/journal.pone.0089473 (PMC3933544; doi:10.1371/journal.pone.0089473)
Supplement: Figure S2 — Comparison of oocyst counts in the infected mosquitoes fed on nitroquine- and buffer-treated mice. (DOC) [file pone.0089473.s002.doc]

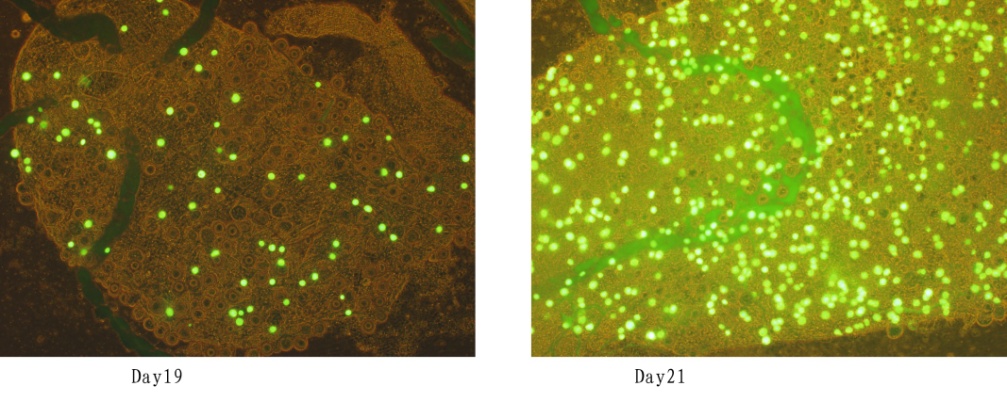

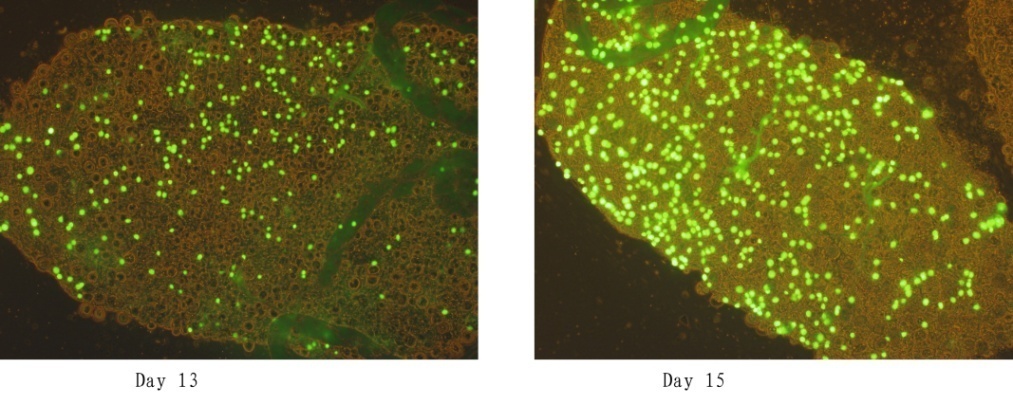


**Figure S2.** Comparison of oocyst counts in the infected mosquitoes fed on nitroquine- or buffer-treated mice. Female mice were inoculated with *P. yoelii (*By265-GFP) parasitized erythrocytes from a donor mouse. When the gametocitaemia reached 1%, nitroquine (ID) or buffer (IB) was administered. Gametocytaemia and parasite exagellation were confirmed 4 h later prior to mosquito blood feeding. The oocyst number in midgut was measured at 10 days after the blood meal. Each assay was done with at least 25 mosquitoes, and the data represent three independent experiments. Left panel: representative fluorescence microscopy images of the GFP-expressing oocysts in midgut of the ID (upper) and IB (bottom) groups. Right panel: statistic analysis of the oocyst numbers in the two groups. Difference of infection intensity between groups was analyzed by paired sample t-test (Prism 6.01, GraphPad Software, Inc.).
